# The Association Between Ascorbate and the Hypoxia-Inducible Factors in Human Renal Cell Carcinoma Requires a Functional Von Hippel-Lindau Protein

**DOI:** 10.3389/fonc.2018.00574

**Published:** 2018-11-30

**Authors:** Christina Wohlrab, Margreet C. M. Vissers, Elisabeth Phillips, Helen Morrin, Bridget A. Robinson, Gabi U. Dachs

**Affiliations:** ^1^Mackenzie Cancer Research Group, Department of Pathology and Biomedical Science, University of Otago, Christchurch, New Zealand; ^2^Centre for Free Radical Research, Department of Pathology and Biomedical Science, University of Otago, Christchurch, New Zealand; ^3^Cancer Society Tissue Bank, University of Otago, Christchurch, New Zealand; ^4^Canterbury Regional Cancer and Haematology Service, Canterbury District Health Board, Christchurch Hospital, Christchurch, New Zealand

**Keywords:** clear cell renal cell carcinoma, papillary renal cell carcinoma, HIF-1, HIF-2, vitamin C, VHL

## Abstract

Hypoxia-inducible transcription factors (HIFs) drive angiogenesis and cancer cell growth, contributing to an aggressive tumor phenotype. HIF-α protein levels and activity are controlled at the post-translational level by HIF hydroxylases. Hydroxylated HIF-α is recognized by the von Hippel Lindau (VHL) tumor suppressor and targeted for degradation. The HIF hydroxylases are members of the iron and 2-oxoglutarate-dependent dioxygenases, which require ascorbate as cofactor for activity. Clear cell renal cell carcinomas (ccRCC) harbor mutations in the *VHL* gene, whereas papillary RCC (pRCC) have a functional VHL. These natural occurring *VHL* variants in RCC enable the testing, in clinical samples, of the hypothesis that ascorbate modulates HIF-α levels through its role as a cofactor for the HIF hydroxylases. We measured ascorbate, HIF-1α, and HIF-2α protein and HIF downstream targets BNIP3, CA9, cyclin D1, GLUT1, and VEGF (combined to generate the HIF pathway score) in VHL-defective ccRCC (*n* = 73) and VHL-proficient pRCC human tumor tissue (*n* = 41). HIF and ascorbate levels were increased in ccRCC and pRCC tumors compared to matched renal cortex. HIF-1 and total HIF pathway activation scores were decreased with higher ascorbate in pRCC tumors (Spearman *r* = −0.38, *p* < 0.05 and *r* = −0.35, *p* < 0.05). This was not evident for ccRCC tumors. In mechanistic studies *in vitro*, ascorbate influenced HIF-1 activity in VHL-proficient, but not VHL-defective ccRCC cells. Our results indicate that ccRCC, which lacks a functional VHL, does not respond to ascorbate-mediated modulation of the HIF response. This contrasts with the demonstrated association between ascorbate content and the HIF pathway observed in pRCC and other tumors with a functional VHL. The results support a role for ascorbate as a modulator of HIF activity and tumor aggression in cancer types with a functional hypoxic response.

## Introduction

The hypoxia-inducible factors (HIFs) are heterodimeric pro-survival transcription factors consisting of oxygen-sensitive regulatory α subunits (HIF-1α, HIF-2α, or HIF-3α) and a stably expressed HIF-1β subunit (1, 2). The HIFs regulate genes involved in glycolysis, angiogenesis, and cell life and death pathways, and influence tumor growth, adaptation to the microenvironment, and resistance to chemo- and radio-therapy (2). Under physiological conditions HIF-α protein levels are tightly regulated by post-translational hydroxylation by enzymes belonging to the family of Fe(II) and 2-oxoglutarate-dependent dioxygenases. These hydroxylases require oxygen, 2-oxoglutarate, ascorbate, and iron for activity (3). Hydroxylation of two proline residues on the HIF-α subunits by proline hydroxylases (PHDs) targets the protein for degradation via the von Hippel-Lindau (VHL) ubiquitin ligase complex (4, 5). Hydroxylation of an asparagine in the C-terminal transactivation domain of HIF-α by the enzyme factor-inhibiting HIF (FIH) leads to repression of transcriptional activity by inhibiting interaction with the p300 coactivator. Inadequate supply of the hydroxylases with either of the two substrates oxygen or 2-oxoglutarate, as well as absence of the essential cofactors ascorbate or iron, impairs activity, leading to increased HIF stabilization and activation (4, 5). Whereas, the dependence on oxygen, 2-oxoglutarate and iron availability are well characterized, less is known about the effect of variable ascorbate concentrations on activity of the HIF hydroxylases. A number of studies have demonstrated that low intracellular ascorbate concentration exacerbates HIF-1 activation in cancer and normal cells (5, 6). Specifically, we and others have demonstrated an inverse association between tumor ascorbate levels and HIF-1 activation in clinical samples from patients with colorectal, endometrial, and thyroid cancer (7–9). Increased tumor ascorbate content was associated with significantly improved disease-free survival for colorectal cancer patients (8).

Ascorbate is widely used as a complementary therapy by cancer patients (10), without robust evidence for efficacy, although a number of reports suggest a benefit in animal studies (11, 12). Ascorbate has therefore been proposed as a potentially useful adjuvant therapy for cancer [reviewed in Wilson et al. (13)], but there is currently debate concerning the proposed mechanism of action [reviewed in Vissers and Das (14)]. Moderation of HIF-1 activation appears likely, although current data only demonstrates an association and not causation (7–9). To date, there are no reports describing the relationship between HIF-2 activity and ascorbate *in vivo*.

Therefore, the aim of this study was to investigate the mechanism of the relationship between ascorbate and HIF activation in cancer cells. To do this we have chosen to analyze this mechanism in renal cell carcinoma (RCC). RCC comprises a number of histological types, with the most common and aggressive being clear cell RCC (ccRCC), accounting for 70% of cases, and papillary RCC (pRCC) with 10–15% of cases (15). About 90% of ccRCC tumors harbor biallelic alterations in the *VHL* tumor suppressor gene, while pRCCs retain a functional VHL protein (16, 17). Loss of VHL function in ccRCC abrogates the controlled degradation of HIF-α leading to constant stabilization and HIF activation, even in adequately oxygenated tissue (18).

The presence of natural occurring *VHL* variants in RCC allowed us to investigate the role of VHL in the response of HIFs to ascorbate. We hypothesize that HIF activation would be unaffected by tumor ascorbate levels in ccRCC, but not in pRCC. To test this, we have analyzed banked tumor tissue along with matched kidney cortex of patients with ccRCC and pRCC for ascorbate content and both HIF-1 and HIF-2 activation. Protein levels of HIF-1α and HIF-2α were measured along with the HIF-1 downstream targets BCL2/adenovirus E1B 19 kDa protein-interacting protein 3 (BNIP3) and carbonic anhydrase 9 (CA9), the HIF-2 target cyclin D1, and the common targets glucose transporter 1 (GLUT1) and vascular endothelial growth factor (VEGF). Ascorbate levels and HIF-pathway activity were compared to clinico-pathological characteristics, and patient survival was followed for 10 years.

## Materials and methods

### Patient samples

Tissue samples were donated to the Cancer Society Tissue Bank (CSTB) Christchurch (HDEC Registration 16STH92) upon surgical removal of the tumor. Donors gave informed written consent for the use of their samples and for access to medical records for research. Ethical approval for this study was granted by the University of Otago Human Ethics committee (reference code H14/020) and samples were approved for use in this study by the CSTB board. In total, samples from 73 patients with ccRCC and 41 with pRCC undergoing nephrectomy between 1998 and 2014 were collected. Matched renal tissue from the cortex was obtained from each patient as part of the resection. The tissue samples were flash frozen in liquid nitrogen within 1 h of surgery and stored at −80°C. Clinico-pathological data and patient characteristics were collected and follow-up acquired from pathology reports and medical records (Tables 1, 2).

**Table 1:** Patient demographics and tumor characteristics for papillary RCC.

**Clinical parameter**	**pRCC number (%)**
Total *n*	41 (100)
**GENDER**	
Female	15 (37)
Male	26 (63)
**AGE**^a^	
< 65 years	17 (41)
≥65 years	24 (59)
**ETHNICITY**	
European	38 (93)
Māori	1 (2)
Other	2 (5)
**FUHRMAN GRADE**	
1	3 (7)
2	20 (49)
3	18 (44)
**SUBTYPE**	
1	14 (34)
2	21 (51)
ND	6 (15)
**PATHOLOGICAL STAGE**	
pT1	28 (68)
pT2	7 (17)
pT3	6 (15)
**NECROSIS**	
Yes	22 (54)
No	19 (46)
**METASTASIS**	
Yes	4 (10)
No	37 (90)
**LYMPH/VASCULAR Invasion**	
Yes	1 (2)
No	40 (98)

a *mean age for pRCC is 66 years. pT, pathological tumor stage; ND, no data*.

**Table 2:** Patient demographics and tumor characteristics for clear cell RCC.

**Clinical parameter**	**ccRCC number (%)**
Total n	73 (100)
**GENDER**	
Female	35 (48)
Male	38 (52)
**AGE**^a^	
< 65 years	37 (51)
≥65 years	36 (49)
**ETHNICITY**	
European	64 (88)
Māori	5 (7)
Other	4 (5)
**FUHRMAN GRADE**	
1	18 (25)
2	18 (25)
3	20 (27)
4	17 (23)
**PATHOLOGICAL STAGE**	
pT1	41 (56)
pT2	7 (10)
pT3	24 (33)
ND	1 (1)
**NECROSIS**	
Yes	24 (33)
No	49 (67)
**METASTASIS**	
Yes	19 (26)
No	54 (74)
**LYMPH/VASCULAR INVASION**	
Yes	21 (29)
No	52 (71)

a *mean age for ccRCC is 62 years. pT, pathological tumor stage; ND, no data*.

### Tissue preparation

Frozen tissue samples were ground to a fine powder in liquid nitrogen using a mortar and pestle chilled on dry ice, and homogenized with 10 mM potassium phosphate buffer (pH 7.4) for DNA isolation, ascorbate extraction, and VEGF ELISA, or with RIPA buffer [50 mM Tris (pH 8), 150 mM NaCl, 1% NP-40, 0.5% sodium deoxycholate, 0.1% SDS (all chemicals from Sigma-Aldrich, St Louis, USA), Complete^TM^ Protease Inhibitor Cocktail (Roche, Basel, Switzerland)] for immunoblot analysis.

### DNA content

DNA content was measured as an indication of the cellular content of the tissue sample. The tissue homogenate was diluted 1:25 with 10 mM potassium phosphate buffer (pH 7.4) and cellular DNA was released by repeated freezing and thawing, followed by sonication for 15 s. Propidium iodide (Life technologies, Carlsbad, USA) was added to a final concentration of 1 mg/ml and DNA was measured by fluorescence at 544–590 nm using black 96-well microplates. DNA concentration was calculated relative to a standard series of 1.25–40 μg/ml purified calf thymus DNA (Invitrogen, Carlsbad, USA).

### Ascorbate content

Ascorbate was immediately stabilized in the homogenized tissue extract by adding an equal volume of 0.54 M perchloric acid containing 50 mM diethylenetriaminepentaacetic acid. Precipitated protein was pelleted by centrifugation (10,000 rpm for 5 min at 4°C) and ascorbate was measured in the supernatant. Samples were reduced with tris(2-carboxyethyl)phosphine (10 mg/ml) for 3 h at 4°C to yield total ascorbate. The Ultimate 3000 HPLC system in reversed phase separation mode coupled to an electrochemical detector was used to measure ascorbate as described previously (4, 5) and the concentration was assessed relative to standards ranging from 1.25 to 40 μM sodium-L-ascorbate (all chemicals from Sigma-Aldrich, St Louis, USA) made fresh for each run.

### Western blotting

Tissue homogenates were standardized to 0.5 μg DNA per well, for cell lysates 20 μg protein was loaded per well. Proteins were separated on 4–12% Bis-Tris Plus SDS gels and transferred to 0.45 μm polyvinylidene difluoride membranes (Life technologies, Carlsbad, USA). The same positive control (20 μg protein of 1% O_2_ hypoxia-treated T24 cell lysate) was run on each gel to normalize signals between the blots. Membranes were incubated overnight at 4°C with primary antibodies against HIF-1α (1/800, BD Biosciences, BD610958), HIF-2α (1/400, R&D Systems, AF2997), BNIP3 (1/1000, R&D Systems, AF4147), CA9 (1/200, R&D Systems, AF2188), cyclin D1 (1/10,000, Abcam, ab134175), GLUT1 (1/1000; Abcam, ab32551) or ß-actin (1/10,000, Sigma, A5316), and for 1 h at room temperature with secondary anti-goat, anti-mouse or anti-rabbit horseradish peroxidase-conjugated antibodies (1/5000, DAKO) as appropriate. B-actin was used as loading control. Protein bands were detected using the ECL Prime Western Blotting Detection Reagent (GE Healthcare, Chicago, USA) and the Alliance 4.7 imaging system, and quantified with ImageJ software (19).

### VEGF elisa

VEGF protein levels in tissue lysates were measured in the same samples prepared for DNA extraction using the human VEGF DuoSET ELISA Development Kit (R&D Systems, DY293B) according to the manufacturer's instructions.

### Cell culture

The human ccRCC cell lines Caki-1 (HTB-46, VHL-proficient) and Caki-2 (HTB-47, VHL-defective) were obtained from the American Type Culture Collection (ATCC; Manassas, VA, USA). Cells were cultured in McCoy's 5A (modified) Medium with 10% fetal bovine serum and 1% Antibiotic-Antimycotic solution (all from Life Technologies, Carlsbad, CA, USA) at a temperature of 37°C, a relative humidity of 95% and an atmosphere containing 5% CO_2_. Cells were regularly tested for mycoplasma by PCR.

### *In vitro* experiments

HIF-1α was stabilized in the cell lines by exposure to 5% O_2_. Cells were seeded into 6-well plates, grown to 80% confluence in air and placed in a H35 Hypoxystation (Don Whitley Scientific Limited, Shipley, UK) for 8 h at 37°C, gassed with a defined oxygen concentration with a balance of N_2_ and 5% CO_2_. To evaluate the effect of ascorbate on stabilization of HIF-1α, cells were pre-loaded with 500 μM ascorbate (Sigma-Aldrich) for 16 h prior to hypoxia treatment. These conditions result in saturating intracellular levels of ascorbate (4). Cells were lysed on the plates in RIPA buffer using a cell scraper and further processed by aspirating through a 27 G needle with a syringe and centrifugation at 12,000 g for 5 min at 4°C. Supernatants were used for Western bot analysis.

### Statistical analysis

Data analysis was performed in GraphPad Prism 5. The Shapiro-Wilk test was used for normality testing. Statistical significance between renal cortex and tumor data was tested with the non-parametric Wilcoxon matched-pairs signed rank test. The Mann Whitney *U*-test was used for comparing unpaired data. Correlations were determined with the Spearman's correlation coefficient. Accumulation of ascorbate in tumor compared to renal cortex tissue was evaluated with the Wilcoxon signed rank test. Survival analysis was performed using log-rank test, and Chi^2^ or Fisher's exact test were used to compare categorical variables. Values of *p* < 0.05 were considered significant; ^*^p < 0.05; ^**^p < 0.01; ^***^p < 0.001.

## Results

### Patient demographics

Samples from 114 kidney cancer patients were included in the study, representing 41 patients with papillary RCC Fuhrman grade 1–3 (Table 1), and 73 patients with clear cell RCC grade 1–4 (Table 2). RCCs ranged from small tumors confined to the kidney (pT1) to large invasive tumors (pT3). Patients in the pRCC cohort had a mean age at diagnosis of 66 years, most were male (63%) and NZ European (93%), with the remainder of the population being either NZ Māori or other (Pacific Islander, Asian) (Table 1). Most pRCC tumors were classified as type 2 (53%) vs. type 1 (34%); patients with type 2 have been reported to have a poorer prognosis than those with type 1 (20). Patients in the ccRCC cohort were on average 62 years old at diagnosis, a slim majority was male (52%) and the majority were NZ European (88%) (Table 2).

### Ascorbate content in RCC patient samples

**Figure 1:**
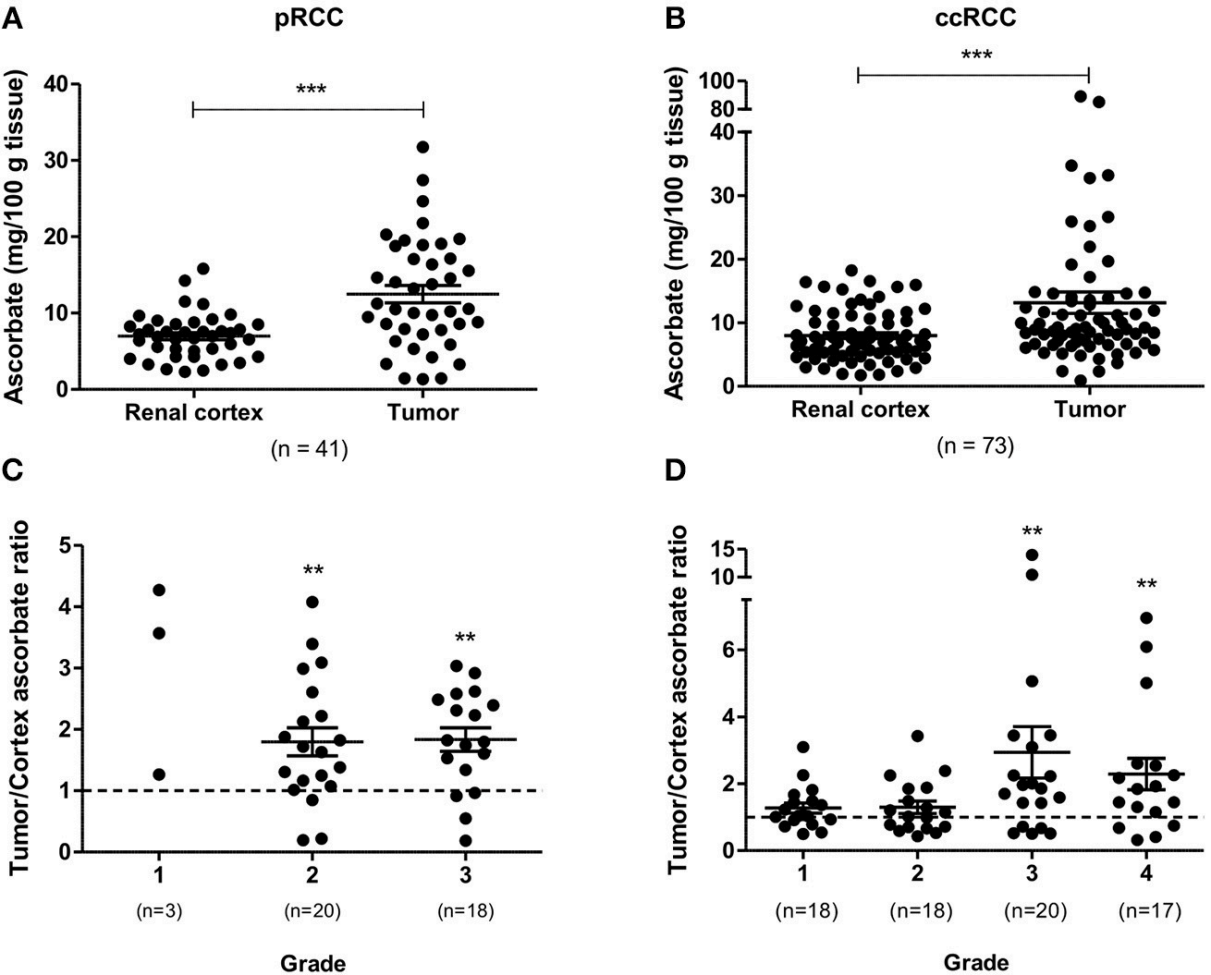
Ascorbate content of pRCC and ccRCC samples compared to renal cortex tissue. Tumor ascorbate levels are increased compared to renal cortex of the same patient for both pRCC (*n* = 41) and ccRCC (*n* = 73) as determined by Wilcoxon matched-pairs signed rank test **(A,B)**. Tumor to renal cortex ascorbate ratio by Fuhrman grade **(C,D)**. Tumor ascorbate content is significantly higher than matched renal cortex in Fuhrman grade 2 and 3 pRCCs and grade 3 and 4 ccRCCs (Wilcoxon signed rank test). Error bars represent mean ± SEM; ^**^p < 0.01, ^***^p < 0.001.

Total tumor tissue ascorbate content in patient samples, measured using HPLC with electrochemical detection, was comparable in pRCC and ccRCC (mean ascorbate concentration of 12.48 and 13.15 mg/100 g tissue, respectively). Tumors had significantly increased ascorbate levels compared to matched renal cortex for both pRCC (*p* < 0.001; Figure 1A) and ccRCC (*p* < 0.001; Figure 1B), indicating increased uptake by the cancer cells relative to the cells present in the renal cortex. The resultant tumor/cortex tissue ascorbate ratio reflects the relative ability of the tumor to accumulate ascorbate from the circulation. In pRCC patients, this ratio indicated an elevated uptake of ascorbate in tumors of grade 2 and 3 above kidney cortex (*p* < 0.01, *p* < 0.01, respectively; Figure 1C). Absolute pRCC tumor ascorbate content was not associated with grade, stage or other patient demographics (Table S1).

In the ccRCC patient cohort, the tumor/cortex ascorbate ratio was significantly increased in grades 3 and 4 (*p* < 0.01, respectively), but not in grades 1 and 2 (Figure 1D). No associations of absolute tumor ascorbate content and patient characteristics or histopathological data were observed (Table S2).

### HIF and target genes in RCC patient samples

**Figure 2:**
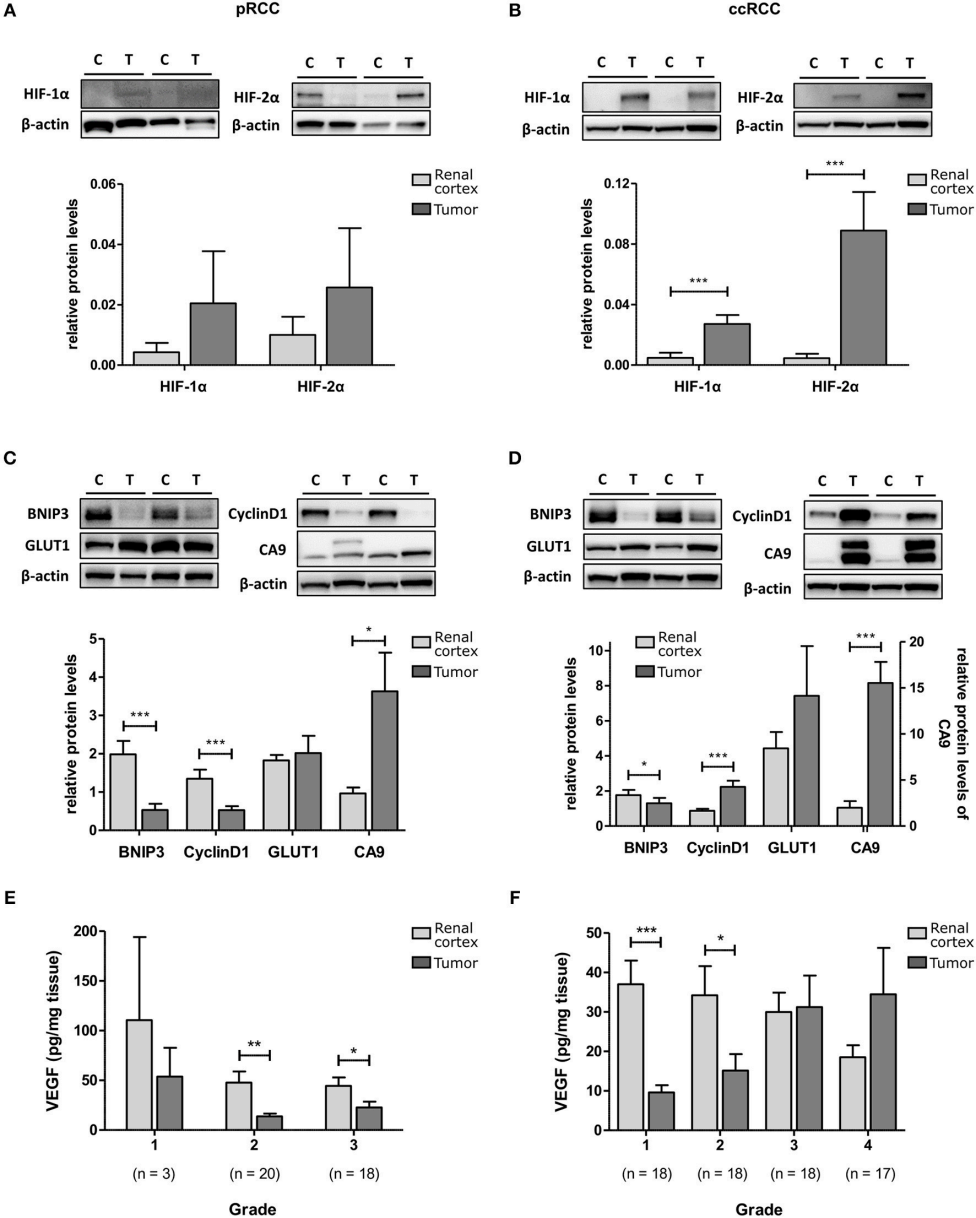
HIF-1 and HIF-2 activation in pRCC and ccRCC samples compared to renal cortex tissue. Representative Western Blots for renal cortex (C) and tumor (T) tissue for pRCC (*n* = 41, left panel) and ccRCC (*n* = 73, right panel) are shown for HIF-1α and HIF-2α **(A,B)**, and their downstream targets BNIP3, cyclin D1, Glut-1 and CA9 **(C,D)**. β-actin was used as a loading control. The example blots in **(B)** show the same blot probed sequentially after stripping of previous primary and secondary antibodies. Bar charts show the band density of the respective proteins referenced to a control sample (Hypoxia treated T24 cell lysate) which was loaded on each gel. VEGF content of RCC and cortex tissue samples, as measured by ELISA, is reduced in tumor tissue of all grades for pRCC **(E)** and low grade ccRCC **(F)** with tendency to be elevated in high grade ccRCC, compared to cortex tissue. Light gray, renal cortex tissue; dark gray, tumor tissue. Statistical significance was assessed with the Wilcoxon matched-pairs signed rank test. Data represent mean + SEM; ^*^p < 0.05; ^**^p < 0.01; ^***^p < 0.001.

To determine HIF pathway activity in RCC tumors, protein levels of HIF-1α, and HIF-2α and their selected downstream targets were measured with Western blot or ELISA. HIF-1α and HIF-2α were elevated in both pRCC and ccRCC tumor types compared to kidney cortex tissue with the difference reaching significance in ccRCC (*p* < 0.001; Figures 2A,B). However, the expression levels of the HIF downstream targets showed a different pattern. In pRCC tumors, expression of the HIF-1 target BNIP3 and the HIF-2 target cyclin D1 were significantly decreased relative to renal cortex tissue, and GLUT1 expression was at similar levels (Figure 2C). In ccRCC, there was a significant increase in the expression of cyclin D1 and a trend for increased levels of GLUT1 compared with the expression measured in renal cortex tissue (Figure 2D). CA9, a downstream target for HIF-1 but not HIF-2, was significantly elevated in both RCC sub-types (Figures 2C,D). VEGF protein, a target gene for both HIF-1 and HIF-2, was found to be expressed at lower levels than in cortex tissue in all grades of pRCC (Figure 2E) while in ccRCC, levels were only decreased in lower grade tumors compared to matched renal cortex tissue and increased in the tumors with increasing grade (*p* < 0.05; Figure 2F).

In pRCC tumors, analysis of the HIF pathways vs. patient characteristics showed an association between higher HIF-2 pathway scores and the more aggressive subtype 2 (*p* < 0.05; Table S1). Amongst ccRCC patients, tumors of lower stage had lower HIF-1 pathway and HIF-2 pathway scores (*p* < 0.05 and *p* < 0.05, respectively; Table S2). No other association of patient characteristics or histopathological data with tumor HIF pathway activity were observed (Tables S1, S2).

### Relationship between ascorbate content and HIF activity in RCC tumors

**Figure 3:**
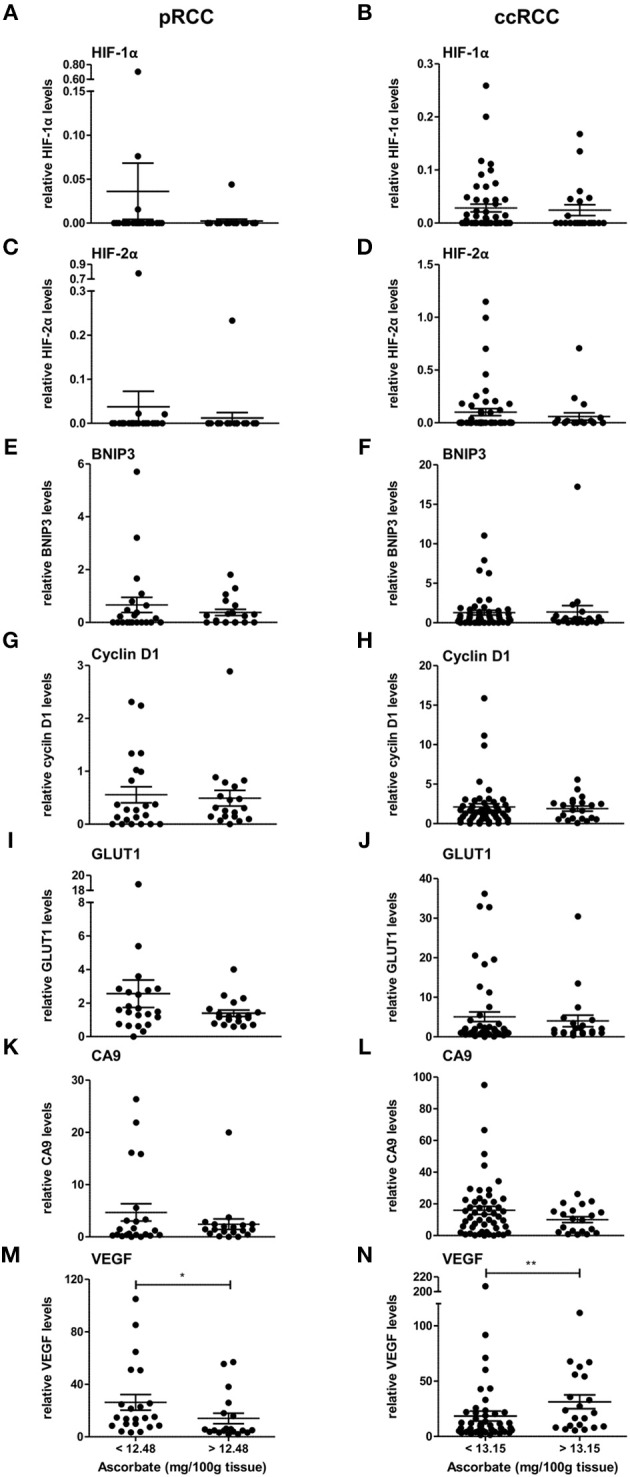
Expression of HIF pathway proteins of pRCC and ccRCC tumors in relation to tissue ascorbate content. Bar plots show relative expression values of HIF-1α **(A,B)**, HIF-2α **(C,D)**, BNIP3 **(E,F)**, cyclin D1 **(G,H)**, GLUT1 **(I,J)**, CA9 **(K,L)**, and VEGF **(M,N)** of pRCC (*n* = 41, left panel) and ccRCC tumors (*n* = 73, right panel) divided by the respective mean ascorbate content. There was no significant difference between the groups, except for VEGF. However, pRCC tumors with less tissue ascorbate levels tended to have increased HIF pathway protein levels. Differences between the groups where evaluated with the Mann-Whitney test. Data represent mean + SEM; ^*^p < 0.05, ^**^p < 0.01.

The expression levels of HIF-1α, HIF-2α, and downstream genes were assessed according to ascorbate content in the tumor tissue. As shown in Figure 3, there was a tendency for levels of all seven proteins to be decreased in pRCC samples with ascorbate levels above the mean, although the difference did not reach significance for the individual proteins except for VEGF, which was significantly decreased in tumors with ascorbate levels above the mean (*p* < 0.05; Figure 3M). Analysis of associations between ascorbate and HIF pathway protein levels showed negative *r*-values for most factors but no significant correlations (*P*-values ranging from 0.1 to 0.9 as shown in Table 3).

**Table 3:** Spearman correlations between tumor ascorbate content and HIF pathway proteins and HIF pathway scores for pRCC (*n* = 41) and ccRCC patients (*n* = 73).

Ascorbate^*^		**HIF-1α**	**HIF-2α**	**GLUT1**	**CA9**	**BNIP3**	**Cyclin D1**	**VEGF**	**HIF-1 pathway score**	**HIF-2 pathway score**	**HIF pathway score**
**pRCC**	r	−0.233	−0.237	−0.223	0.028	−0.003	0.065	−0.256	−**0.378**	−0.151	−**0.346**
	p	0.143	0.136	0.162	0.864	0.986	0.688	0.106	<**0.05**	0.348	<**0.05**
**ccRCC**	r	−0.027	0.045	0.006	0.047	0.078	−0.010	**0.321**	0.073	0.081	0.044
	p	0.825	0.707	0.957	0.700	0.513	0.932	<**0.01**	0.542	0.497	0.714

*Significant correlations are shown in bold*.

* *(mg/100 g tissue)*.

In contrast, the same pattern was not seen in ccRCC tumors in which protein levels were unaffected by differences in ascorbate content (Figure 3, Table 3), with the exception of VEGF, which showed a significant increase in tumors with above the mean ascorbate content (*p* < 0.01; Figure 3N).

**Figure 4:**
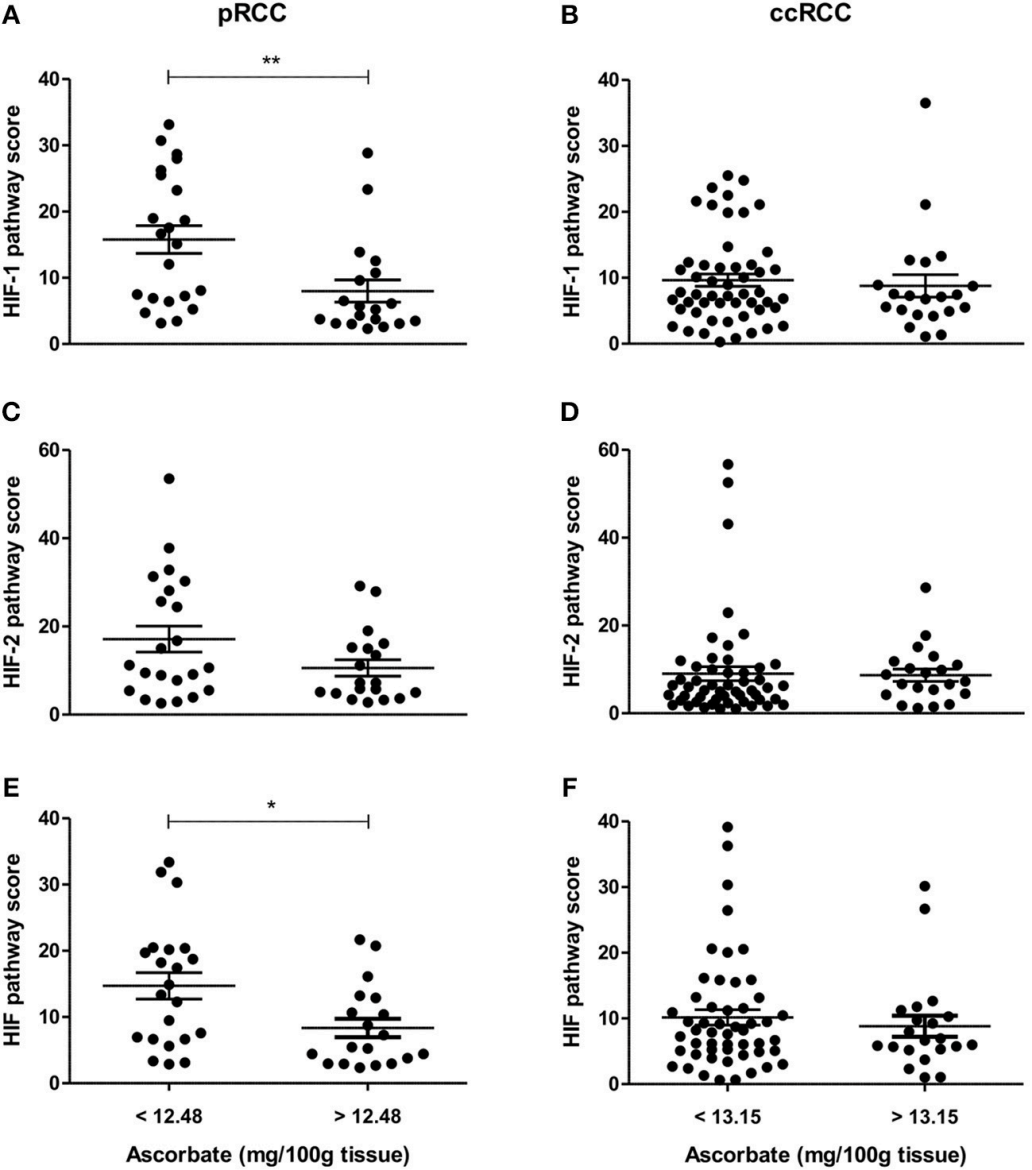
HIF pathway scores of pRCC and ccRCC tumors in relation to tissue ascorbate content. Scatter plots show HIF-1 **(A,B)**, HIF-2 **(C,D)**, and HIF pathway scores **(E,F)** of pRCC (*n* = 41, left panel) and ccRCC tumors (*n* = 73, right panel) divided by the mean ascorbate content. Differences between the groups where evaluated with the Mann-Whitney test. Data represent mean + SEM; ^*^p < 0.05, ^**^p < 0.01.

To evaluate the impact of ascorbate on HIF transcription factor activity, a global HIF pathway score was assigned to each tumor sample by normalizing the relative expression values for each protein as percent expression of the highest sample. The HIF-1 pathway score combined HIF-1α, BNIP3, CA9, GLUT1, and VEGF scores, the HIF-2 pathway score combined HIF-2α, cyclin D1, GLUT1, and VEGF scores, and the total HIF pathway score combined all measures. For the pRCC cohort, there was a significant negative correlation between tumor ascorbate content and HIF-1 and total HIF pathway scores (*r* = −0.378, *p* < 0.05 and *r* = −0.346, *p* < 0.05, respectively; Table 3). Samples with above mean tumor ascorbate content (12.48 mg/100 g tissue) had significantly lower HIF-1 and total HIF pathway scores when compared to tumors with below median ascorbate concentration (*p* < 0.01 and *p* < 0.05, respectively; Figures 4A,E). HIF-2 pathway scores showed the same trend, but did not reach significance (Figure 4C and Table 3).

In contrast, there was no association between ascorbate levels and HIF-1, HIF-2 or the combined HIF pathways in ccRCCs (Figures 4B,D,F, Table 3).

### Patient survival data

Patients were followed up for 10 years following surgical removal of the affected kidney, and tumor ascorbate levels were compared against rates of recurrence, metastases, disease-specific and all-cause mortality. Median time of follow-up was 9.1 years for pRCC and 8.8 years for ccRCC patients. Nephrectomy is an effective treatment option for early stage RCC and median disease-free and overall survival was not reached.

At follow-up, 71% of patients with pRCC were alive, 90% were disease-free and 2 patients had died from the disease. Four patients (10%) developed metastatic disease after surgery (lung and bone), and five (12%) were diagnosed with a new primary tumor. Among ccRCC patients, 69% of patients were alive, 10 patients (14%) had died from ccRCC, 12 (17%) from other causes, and 77% were disease-free. One patient had a local recurrence, sixteen (23%) developed metastatic disease (lung, bone, liver, adrenal glands, brain, contralateral kidney, breast, and colon), and six (8%) had a new primary tumor. Two ccRCC patients were excluded from survival analysis as they had metastasis at time of surgery.

For analysis of the effect of tumor ascorbate on survival, patients were split into two groups by their mean values for tumor ascorbate content, or HIF-1 and HIF-2 pathway scores, respectively. There was no significant association of ascorbate content or HIF pathway activity with 10-year all-cause mortality in patients with either pRCC or ccRCC (Table S3). Based on four patients who developed metastasis, patients with pRCC and higher tumor ascorbate content had worse disease-free survival (*p* < 0.05, Table S3). In ccRCC patients with higher HIF-2 pathway scores, all-cause mortality and disease-free survival tended to be worse, although significance was not reached (*p* = 0.063 and *p* = 0.092, respectively; Table S3).

### In vitro

To directly test whether ascorbate has an effect on hypoxia-induced HIF pathway protein levels, ccRCC cells were pre-loaded with saturating levels of ascorbate in normoxic culture conditions (21% O_2_) and then subjected to mild hypoxia (5% O_2_).

**Figure 5:**
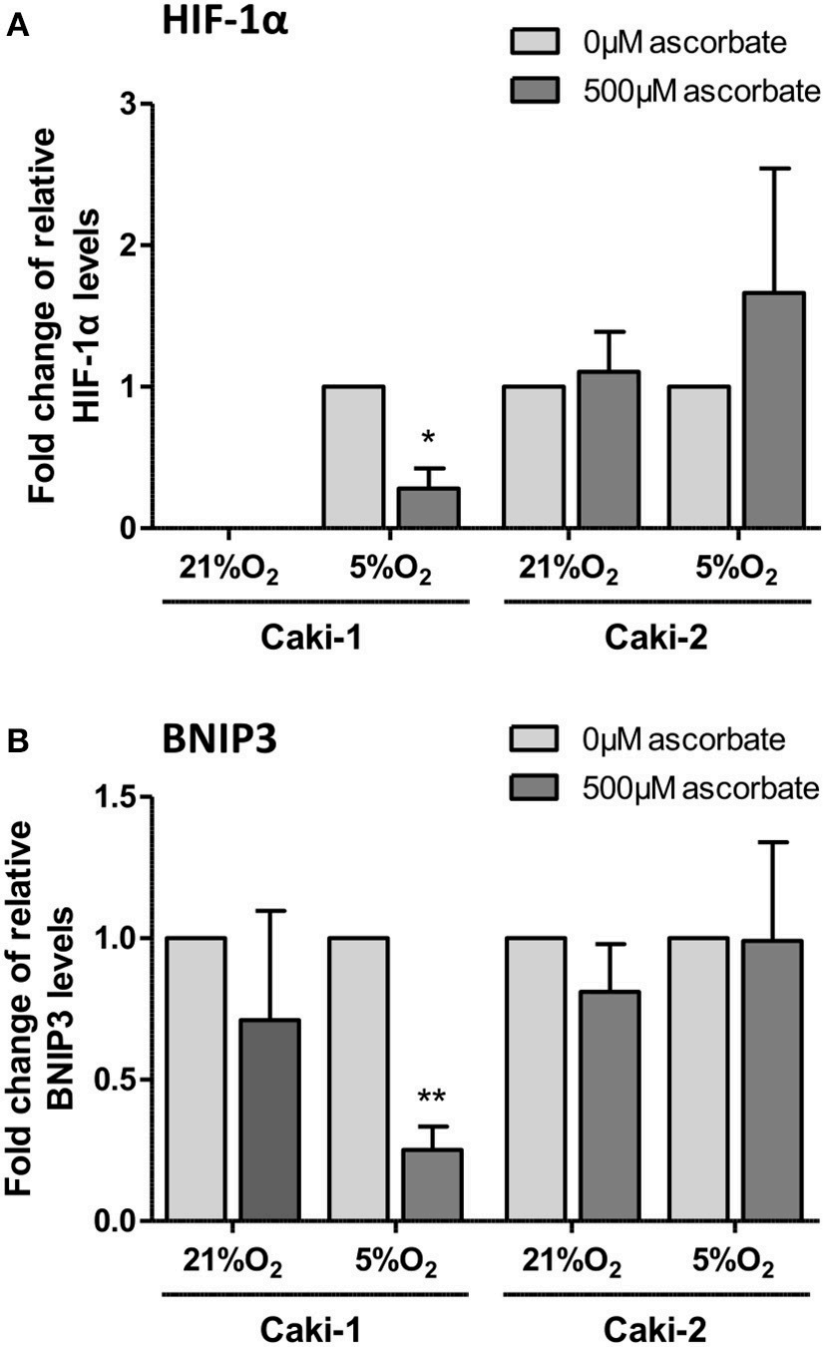
HIF pathway expression in ccRCC cell lines in response to ascorbate. Caki-1 (VHL-proficient) and Caki-2 (VHL-defective) cells were pre-loaded with 500 μM ascorbate for 16 h, or left untreated, and then subjected to 21% O_2_ or 5% O_2_ for 8 h. Protein levels of HIF-1α and BNIP3 were evaluated by Western blot analysis with β-actin as loading control. Densitometry analysis shows protein levels standardized to β-actin and relative to untreated cells (0 μM ascorbate) **(A,B)**. Induced expression of HIF-1α and BNIP3 in Caki-1 cells at 5% O_2_ was repressed by ascorbate treatment. No significant changes in response to ascorbate were observed in Caki-2 cells. Differences between the groups were evaluated by paired *t***-**test. Data represent mean +SD from three independent experiments; ^*^p < 0.05, ^**^p < 0.01.

In VHL-proficient Caki-1 cells, HIF-1α was absent at 21% O_2_ and stabilized at 5% O_2_. Ascorbate exposure reduced levels of HIF-1α (*p* < 0.05) and BNIP3 (*p* < 0.01) compared to ascorbate-depleted cells at 5% O_2_ (Figure 5). In VHL-defective Caki-2 cells, HIF-1α was continuously stabilized, and protein levels were unchanged by ascorbate at 21% O_2_ and slightly elevated at 5% O_2_, but this was not significant (Figure 5A). The downstream target BNIP3 was unaffected by ascorbate at all tested conditions in Caki-2 cells (Figure 5B).

## Discussion

Our study in 114 RCC tumors demonstrates an association between the HIF pathway and ascorbate in tumors that is dependent on the presence of a functional VHL tumor suppressor. The HIF-1 pathway and overall HIF pathway scores were reduced in pRCC tumors with above mean ascorbate levels, reflecting a tendency for reduction of all seven hypoxia-regulated proteins tested. In contrast, these associations were completely absent in ccRCC tumors that are characterized by a dysfunctional VHL. In ccRCC cell lines *in vitro*, ascorbate was able to reduce stability and transcriptional activity of HIF-1, but only in cells with a functional VHL tumor suppressor protein.

**Figure 6:**
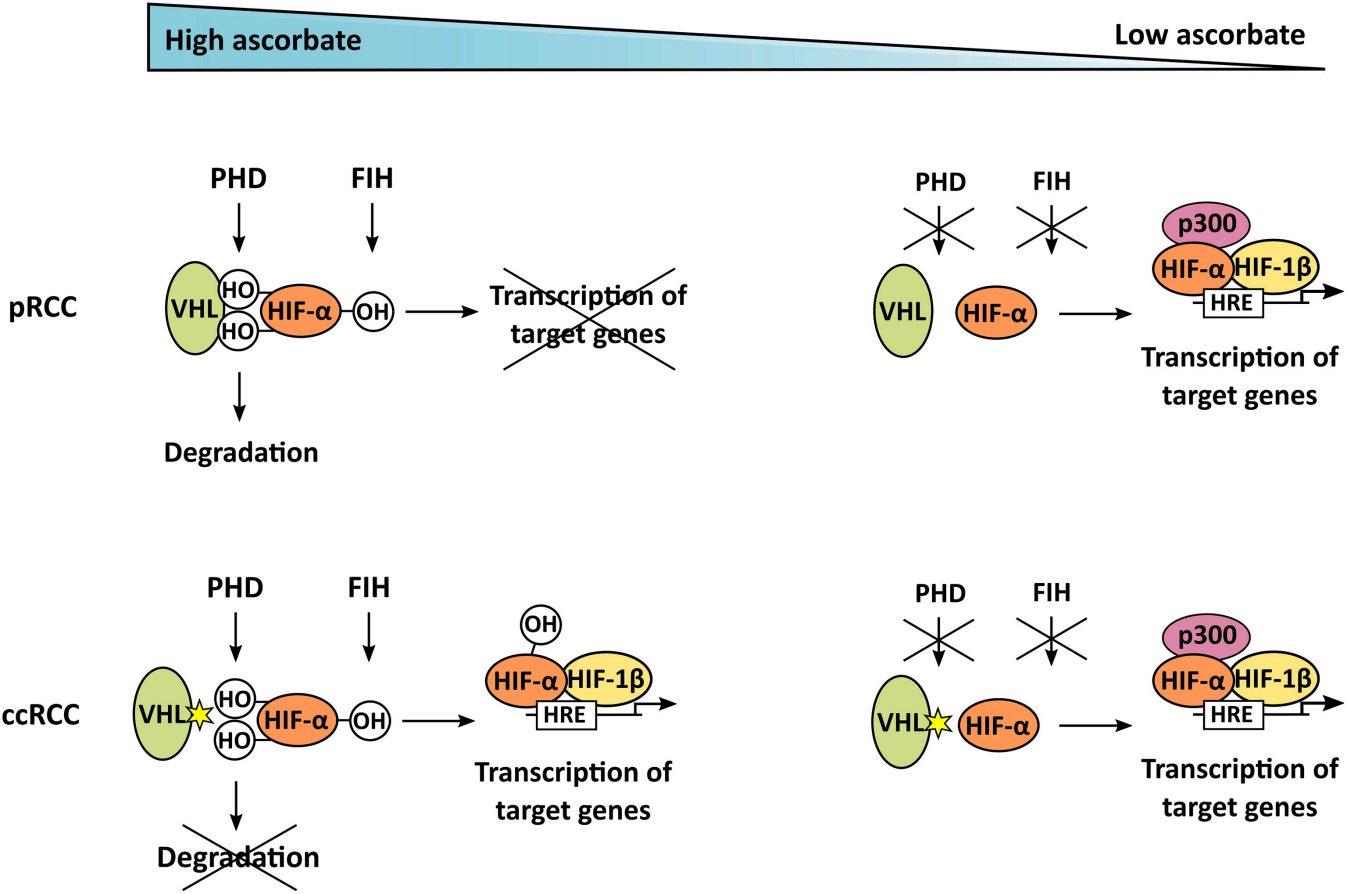
Mechanism of ascorbate-dependent HIF-regulation in pRCC and ccRCC. If enough ascorbate is present, HIF-α is hydroxylated by PHD and FIH. In pRCC, this enables binding of the VHL tumor suppressor, leading to degradation of HIF-α; it also represses target gene transcription. In contrast to that, ccRCC tumors harbor mutations in *VHL*, resulting in uncontrolled accumulation and transcriptional activity of HIF-α, even if ascorbate is abundant and HIF is hydroxylated. In tumors lacking ascorbate, the hydroxylases are not fully functional, HIF-α is stabilized and can trigger target gene transcription after dimerization with HIF-β, and interaction with the p300 coactivator, in both pRCC and ccRCC.

HIF-α proteins and downstream target genes were generally decreased in pRCC samples with the highest ascorbate levels. When considered individually, none of these associations reached statistical significance, but when a HIF pathway score was assigned to each tumor sample by combining the relative expression of HIF-1α or HIF-2α and their target genes, respectively, a significant inverse association between ascorbate and HIF-1 and combined HIF pathways was apparent. We have previously seen this association in endometrial (7), colorectal (8) and breast cancer (unpublished data) and a similar relationship has been reported in thyroid cancer (9). Together with our current results, these data are consistent with the notion that increased ascorbate levels modulate HIF activity by supporting the function of the regulatory hydroxylases, while insufficient supply leads to accumulation and increased transcriptional activity of HIF (Figure 6). However, from the prior analyses, one can only conclude that there is an association between ascorbate levels and HIF activity. They do not directly address the hypothesis, that ascorbate modulates HIF activation by stimulating the HIF hydroxylases.

The current investigation into the two renal cell carcinoma sub-types has allowed us to investigate the potential interaction between ascorbate and the HIF hydroxylases in tumors using ccRCC as an example of naturally occurring tumor mutations, resulting in a dysfunctional VHL protein. This mutation prevents degradation of the hydroxylated HIF-α chains and increases subsequent transcriptional activity of the HIFs in ccRCC, and is therefore independent of the hypoxic status of the tumor, or the availability of other hydroxylase substrates or co-factors, including ascorbate (Figure 6). That there was no association between HIF activity and ascorbate content in ccRCC tumor tissue supports a causative link for this relationship. It remains possible that some of these tumor samples were VHL-proficient, but given that about 90% of all ccRCC cases contain a dysfunctional VHL protein (16, 17) these samples are very unlikely to influence our dataset. It is also possible that HIF activation is regulated by variations in the PHD or FIH enzymes.

Our retrospective analyses of human tumor tissue provides the first evidence from a clinical setting that tissue ascorbate levels could moderate HIF activation through stimulation of the HIF hydroxylases, but only in VHL-proficient tumors. Our data suggest that ascorbate intervention is unlikely to prove beneficial in VHL-defective cancer types such as ccRCC.

Modulation of the HIF pathways in RCC by any means is likely to impact on tumor growth and progression. Differences in effect may reflect activation of either HIF-1 or HIF-2, which have both unique and common target genes and can support or interfere with each other dependent on microenvironment conditions (21). This seems to be especially evident in VHL-defective ccRCC, where HIF-1 can have tumor suppressive effects, whereas HIF-2 appears to be the major player in tumorigenesis (22–24) and is associated with higher grade, stage and poor patient outcome (25, 26). A study comparing multiple cell lines revealed that in VHL-defective ccRCC cells, HIF-1α, and HIF-2α demonstrate prominent transcriptional selectivity for their target genes and even have suppressive effects on each other (23).

Although the role of HIFs in ccRCC has been extensively studied (22, 23), involvement in pRCC is less well understood. We detected significantly lower HIF-2α, BNIP3, cyclin D1, and CA9 protein expression in pRCC compared to ccRCC (all *p* < 0.05), with HIF-1α, GLUT1, and VEGF expressed at similar levels. Although HIF proteins are constitutively synthesized in all cells, in pRCC HIF levels reflect the hydroxylation of the protein and therefore the activity of the PHDs, whereas in ccRCC hydroxylated HIFs will not be degraded and stabilization is independent of the PHD enzymes (Figure 6). However, those pRCC tumors that had ascorbate levels similar to or higher than reported normal levels for the kidney [~10 mg/100 g tissue (27)] had reduced HIF pathway activation, indicating that the hypoxic pathway in pRCC can be modulated by ascorbate.

We found lower expression of VEGF in pRCC and lower grade ccRCCs (grade 1 and 2) compared with kidney cortex tissue. This is in line with another study showing lower expression of the VEGF_165_ and VEGF_189_ isoforms in both pRCC and ccRCC compared to kidney cortex (28). Our finding that VEGF tended to increase in higher-grade ccRCC supports evidence that high VEGF expression is a poor prognostic indicator for patients with ccRCC (25, 26) and is thus a preferential target for therapy (29).

Ascorbate content in RCC was increased in tumors, especially higher grades, compared to adjacent uninvolved tissue (renal cortex) from the same patient, which is in contrast to our previous studies in endometrial and colorectal cancer, which showed decreased levels of ascorbate in higher grade tumors compared to matched normal tissue (7, 8). There are several possible reasons for these differences. RCC tumors arise from the lining of the proximal convoluted tubule, whereas the cortex has a complex composition, containing corpuscles, renal tubules, and cortical collecting ducts. Hence, the cellular composition in our test and control samples were likely very different. In addition, the kidney is a highly specialized organ which filters vast quantities of blood each day, much more than either endometrial or colorectal tissues. The kidney has a unique distribution of sodium-dependent vitamin C transporters (SVCT1 and SVCT2). SVCT1 is situated in the brush-border membrane of proximal tubule cells where it is responsible for maintaining whole body ascorbate levels by mediating renal reabsorption, whereas SVCT2 is expressed basolaterally in all cells in the kidney except the proximal tubule (30–32). Both papillary and clear cell RCC are presumed to derive from the epithelium of the proximal tubule which only expresses SVCT1 (33). To date, protein levels of SVCTs in RCC tumors have not been reported.

RCC affects more than 270,000 individuals worldwide each year (17). It is curable if detected early and treated by partial or radical nephrectomy (29). However, treatment options are limited for patients with advanced stages of the disease, with a 5-year survival of < 10% in patients with distant metastases (34, 41), with the introduction of targeted therapies starting to improve survival rates (35).

Assessing the association between tumor ascorbate levels and survival in this RCC patient cohort was difficult as neither median overall survival nor median disease-free survival was reached. Therefore, although long-term follow-up data was available, analysis was restricted by very few events. Population based studies have also reported that ascorbate did not play a major role in the risk of kidney cancer formation, showing no or only a weak inverse relationship between increased ascorbate intake and RCC risk (36–38), and a weak positive correlation in postmenopausal women (39). Ascorbate data in these studies comes from interviews or questionnaires, and plasma or tissue levels of ascorbate were not measured.

From our current and previous data (7, 8) we propose that elevating tumor levels of ascorbate in pRCC could reduce HIF pathway activation via enhancing HIF hydroxylase enzyme activity (Figure 6). In contrast, we suggest that manipulating ascorbate levels in ccRCC would not affect the hypoxic response and thus would not change tumor aggression that is dependent on this pathway. It is worth noting that there is significant discussion around the best means to deliver ascorbate to tumors, with many advocating for high dose infusions, which result in high extracellular concentrations that may be cytotoxic through other mechanisms such as the extracellular generation of H_2_O_2_ (40). Ascorbate-mediated modulation of HIF activation, on the other hand, relies only on optimal intracellular ascorbate concentrations.

## Conclusion

Our data in pRCC tumors adds further evidence for an association between ascorbate and the HIF-pathway in cancer. The comparison of VHL-defective ccRCC and VHL-proficient pRCC tumors supports the role of ascorbate as a vital cofactor of dioxygenase enzymes that target HIF degradation via VHL. Our data provides no evidence to support ascorbate intervention for ccRCC, which represent the majority of RCC patients.

## Author contributions

GD conceived the study, CW collected the data and composed the manuscript. HM and BR collected medical information. GD, EP, MV and BR edited and refined the manuscript. All authors finalized the manuscript.

### Conflict of interest statement

The authors declare that the research was conducted in the absence of any commercial or financial relationships that could be construed as a potential conflict of interest.

## Abbreviations

RCC: renal cell carcinoma
ccRCC: clear cell RCC
pRCC: papillary RCC
HIF: hypoxia-inducible factor
VHL: von Hippel-Lindau
PHD: proline hydroxylase
FIH: factor inhibiting HIF
BNIP3: BCL2/adenovirus E1B 19 kDa protein-interacting protein 3
CA9: carbonic anhydrase 9
GLUT1: glucose transporter 1
VEGF: vascular endothelial growth factor
CSTB: Cancer Society Tissue Bank
SVCT: sodium-dependent vitamin C transporter.
