# Supplementary material for: The Association Between Ascorbate and the Hypoxia-Inducible Factors in Human Renal Cell Carcinoma Requires a Functional Von Hippel-Lindau Protein
Source: Front Oncol. 2018 Nov 30;8:574. doi: 10.3389/fonc.2018.00574 (PMC6284050; doi:10.3389/fonc.2018.00574)
Supplement: Supplementary file 1 [file Table_1.docx]

Supplementary Material

**The Association between Ascorbate and the Hypoxia-inducible Factors in Human Renal Cell Carcinoma Requires a Functional Von Hippel-Lindau Protein**

**Christina Wohlrab, Margreet CM Vissers, Elisabeth Phillips, Helen Morrin, Bridget A Robinson, Gabi U Dachs***

*** Correspondence:** Gabi Dachs: gabi.dachs@otago.ac.nz

1. **Supplementary Tables**

Supplementary Table S1 Relationship between pRCC patient characteristics (n = 41) and tissue ascorbate content, HIF1 and HIF2 pathway scores.

|  | **Ascorbate (mg/100g tissue)** | |  | **HIF-1 pathway score** | |  | **HIF-2 pathway score** | |  |
| --- | --- | --- | --- | --- | --- | --- | --- | --- | --- |
|  | **<mean** | **>mean** | **p** | **<mean** | **>mean** | **p** | **<mean** | **>mean** | **p** |
| **Age** |  |  | 1 |  |  | 1 |  |  | 1 |
| **<66 years** | 10 | 8 |  | 11 | 7 |  | 11 | 7 |  |
| **≥66 years** | 12 | 11 |  | 14 | 9 |  | 14 | 9 |  |
| **Gender** |  |  | 0.212 |  |  | 0.517 |  |  | 0.517 |
| **Female** | 6 | 9 |  | 8 | 7 |  | 8 | 7 |  |
| **Male** | 16 | 10 |  | 17 | 9 |  | 17 | 9 |  |
| **Ethnicity** |  |  | 0.588 |  |  | 1 |  |  | 1 |
| **European** | 21 | 17 |  | 23 | 15 |  | 23 | 15 |  |
| **Māori** | 0 | 1 |  | 1 | 0 |  | 1 | 0 |  |
| **Other** | 1 | 1 |  | 1 | 1 |  | 1 | 1 |  |
| **Subtype** |  |  | 1 |  |  | 0.100 |  |  | **< 0.05** |
| **Type 1** | 9 | 8 |  | 13 | 4 |  | 14 | 3 |  |
| **Type 2** | 10 | 11 |  | 10 | 11 |  | 9 | 12 |  |
| **Fuhrman grade** |  |  | 0.854 |  |  | 0.393 |  |  | 0.393 |
| **1** | 2 | 1 |  | 1 | 2 |  | 1 | 2 |  |
| **2** | 11 | 9 |  | 14 | 6 |  | 14 | 6 |  |
| **3** | 9 | 9 |  | 10 | 8 |  | 10 | 8 |  |
| **Pathological Stage** |  |  | 0.967 |  |  | 0.750 |  |  | 0.750 |
| **pT1** | 15 | 13 |  | 16 | 12 |  | 16 | 12 |  |
| **pT2** | 4 | 3 |  | 5 | 2 |  | 5 | 2 |  |
| **pT3** | 3 | 3 |  | 4 | 2 |  | 4 | 2 |  |
| **Necrosis** |  |  | 0.756 |  |  | 1 |  |  | 0.757 |
| **Yes** | 11 | 11 |  | 13 | 9 |  | 14 | 8 |  |
| **No** | 11 | 8 |  | 12 | 7 |  | 11 | 8 |  |
| **Lymphovascular invasion** | |  | 0.463 |  |  | 0.390 |  |  | 0.390 |
| **Yes** | 0 | 1 |  | 0 | 1 |  | 0 | 1 |  |
| **No** | 22 | 18 |  | 25 | 15 |  | 25 | 15 |  |

pT, pathological tumor stage

Supplementary Table S2 Relationship between ccRCC patient characteristics (n = 73) and tissue ascorbate content, HIF1 and HIF2 pathway scores.

|  | **Ascorbate (mg/100g tissue)** | |  | **HIF-1 pathway score** | |  | **HIF-2 pathway score** | |  | |
| --- | --- | --- | --- | --- | --- | --- | --- | --- | --- | --- |
|  | **<mean** | **>mean** | **p** | **<mean** | **>mean** | **p** | **<mean** | **>mean** | **p** | |
| **Age** |  |  | 0.602 |  |  | 0.476 |  |  | 0.087 |  |
| **<62 years** | 26 | 8 |  | 23 | 11 |  | 26 | 8 |  |  |
| **≥62 years** | 27 | 12 |  | 23 | 16 |  | 22 | 17 |  |  |
| **Gender** |  |  | 1 |  |  | 0.636 |  |  | 1 | |
| **Female** | 25 | 10 |  | 21 | 14 |  | 23 | 12 |  | |
| **Male** | 28 | 10 |  | 25 | 13 |  | 25 | 13 |  | |
| **Ethnicity** |  |  | 0.403 |  |  | 0.632 |  |  | 0.069 | |
| **European** | 45 | 19 |  | 40 | 24 |  | 39 | 25 |  | |
| **Māori** | 4 | 1 |  | 4 | 1 |  | 5 | 0 |  | |
| **Other** | 4 | 0 |  | 2 | 2 |  | 4 | 0 |  | |
| **Fuhrman grade** |  |  | 0.084 |  |  | 0.474 |  |  | 0.532 | |
| **1** | 15 | 3 |  | 13 | 5 |  | 14 | 4 |  | |
| **2** | 16 | 2 |  | 9 | 9 |  | 12 | 6 |  | |
| **3** | 12 | 8 |  | 14 | 6 |  | 11 | 9 |  | |
| **4** | 10 | 7 |  | 10 | 7 |  | 11 | 6 |  | |
| **Pathological Stage** |  |  | 0.052 |  |  | **< 0.05** |  |  | **< 0.05** | |
| **pT1** | 33 | 8 |  | 31 | 10 |  | 32 | 9 |  | |
| **pT2** | 6 | 1 |  | 2 | 5 |  | 3 | 4 |  | |
| **pT3** | 13 | 11 |  | 12 | 12 |  | 12 | 12 |  | |
| **Necrosis** |  |  | 0.263 |  |  | 1 |  |  | 0.606 | |
| **Yes** | 15 | 9 |  | 15 | 9 |  | 17 | 7 |  | |
| **No** | 38 | 11 |  | 31 | 18 |  | 31 | 18 |  | |
| **Lymphovascular invasion** | |  | 0.248 |  |  | 1 |  |  | 1 | |
| **Yes** | 13 | 8 |  | 13 | 8 |  | 14 | 7 |  | |
| **No** | 40 | 12 |  | 33 | 19 |  | 34 | 18 |  | |

pT, pathological tumor stage

Supplementary Table S3 10-year all-cause mortality and disease-free survival of pRCC (n = 41) and ccRCC patients (n = 71) according to tissue ascorbate content, HIF1 and HIF2 pathway scores by Log-rank test. Significant associations are shown in bold.

|  |  | **10-year all-cause mortality** | | | |  | **10-year disease-free survival** | | | |
| --- | --- | --- | --- | --- | --- | --- | --- | --- | --- | --- |
|  | **Mean** | **<mean** | **>mean** | **HR (95% CI)** | **p** |  | **<mean** | **>mean** | **HR (95% CI)** | **p** |
| **Ascorbate*** | | n (%) | n (%) |  |  |  | n (%) | n (%) |  |  |
| pRCC | 12.48 | 4 (18) | 8 (42) | 0.36 (0.12 - 1.14) | 0.083 |  | 0 (0) | 4 (21) | 0.10 (0.01 - 0.70) | **< 0.05** |
| ccRCC | 13.15 | 16 (31) | 6 (32) | 1.03 (0.40 - 2.61) | 0.958 |  | 10 (19) | 7 (37) | 0.46 (0.16 - 1.35) | 0.158 |
| **HIF-1 pathway score** | |  |  |  |  |  |  |  |  |  |
| pRCC | 12.18 | 9 (36) | 3 (19) | 1.80 (0.56 - 5.78) | 0.322 |  | 2 (8) | 2 (13) | 0.63 (0.08 - 4.69) | 0.651 |
| ccRCC | 9.40 | 12 (27) | 10 (38) | 0.56 (0.23 - 1.38) | 0.209 |  | 10 (22) | 7 (27) | 0.70 (0.25 - 1.94) | 0.491 |
| **HIF-2 pathway score** | |  |  |  |  |  |  |  |  |  |
| pRCC | 14.10 | 9 (36) | 3 (19) | 1.81 (0.56 - 5.77) | 0.319 |  | 2 (8) | 2 (13) | 0.63 (0.08 - 4.69) | 0.651 |
| ccRCC | 8.93 | 12 (26) | 10 (42) | 0.41 (0.16 - 1.05) | 0.063 |  | 9 (19) | 8 (33) | 0.40 (0.14 – 1.16) | 0.092 |

HR, hazard ratio; CI, confidence interval; n (%) describes the number of events and percentage of the total n in the two groups below and above the mean ascorbate values; *mg/100 g ascorbate.
